# Genomic Insights into the Probiotic Potential of Lactic Acid Bacteria Isolated from Tocosh: Traditional Peruvian Fermented Potatoes

**DOI:** 10.3390/ijms27093981

**Published:** 2026-04-29

**Authors:** Vilma Julia Reyes, Marcial Silva-Jaimes, Liz Erika Cruz-Pio, Michel Abanto, Mario Taira, Pablo Ramirez

**Affiliations:** 1 Facultad de Ingeniería en Industrias Alimentarias, Universidad Nacional del Centro del Perú, Av. Mariscal Castilla N° 3909, Huancayo 12006, Peru; 2 Facultad de Industrias Alimentarias, Universidad Nacional Agraria La Molina, Av. La Molina s/n, La Molina, Lima 15024, Peru; misilva@lamolina.edu.pe; 3 Facultad de Ciencias Biológicas, Universidad Nacional Mayor de San Marcos, Calle Germán Amezaga 375 Ciudad Universitaria, Lima 15081, Peru; lcruzp1@unmsm.edu.pe (L.E.C.-P.); mario.taira@unmsm.edu.pe (M.T.); pramirezr@unmsm.edu.pe (P.R.); 4 Scientific and Technological Bioresource Nucleus (BIOREN), Universidad de La Frontera, Temuco 4811230, Chile; mfabanto@gmail.com

**Keywords:** tocosh, genomics, probiotics, fermentation, altitude

## Abstract

Tocosh, an ancestral fermented potato product, relies on spontaneous processes near freshwater springs under extreme high-altitude conditions and represents an underexplored reservoir of microbial diversity with significant potential for the discovery of probiotics. This study provides, for the first time, a comprehensive probiogenomic characterization of 19 lactic acid bacteria (LAB) isolated from tocosh, in the Peruvian Andes, at three distinct altitudes—2992, 3882, and 4451 m above sea level (m.a.s.l.)—using whole genome sequencing (WGS) and bioinformatic profiling. A total of six species were identified: *Lactiplantibacillus plantarum* and *Levilactobacillus brevis* at all three study sites, *Lacticaseibacillus paracasei* and *Lentilactobacillus buchneri* at the lowest altitude (2992 m.a.s.l.), and *Latilactobacillus curvatus* and *Latilactobacillus sakei* at the highest altitudes (3882 and 4451 m.a.s.l.). Our results reveal that the extreme Andean environment is associated with stability in *L. plantarum* (genome sizes from 3.36 to 3.38 Mb) across all altitudinal levels. Functional analysis using CAZymes determined that *L. brevis* and *L. buchneri* act as primary degraders (high percentage of glycosyl hydrolases/carbohydrate binding) while *L. curvatus* and *L. sakei* function as primary builders through exopolysaccharide biosynthesis, likely a cryoprotective adaptation preventing cell damage during cold temperatures at high altitudes. Additionally, *L. sakei* and *L. plantarum* exhibited unique auxiliary activity (AA) enzymes, suggesting an oxidative mechanism to breach recalcitrant starch surfaces. All isolates were confirmed as genomically safe, lacking transferable antibiotic resistance genes and virulence factors. Pathogenic risk potential scores (PPRS) were consistently ≤2.0, fulfilling qualified presumption of safety (QPS) criteria. These findings provide the first genomic characterization of tocosh-associated LAB, establishing a basis for tocosh standardization, enabling the rational design of starter cultures that preserve ancestral traits and ensure microbiological safety in modern food applications.

## 1. Introduction

Potato (*Solanum tuberosum* L.) is a fundamental staple crop in the Andean region. To ensure long-term food security and enhance functional properties, ancestral techniques have been developed, including dehydration for papa seca, lyophilization for chuño, and fermentation for tocosh [1]. Tocosh, a product of natural potato fermentation, represents both a biotechnologically significant food and cultural heritage deeply embedded in Andean communal practices [2]. Predominantly produced in the Peruvian highlands—specifically Ancash, Huanuco and Junin—the process leverages diverse potato varieties and specific ecological conditions conducive to spontaneous fermentation [3]. During its traditional fermentation process near freshwater springs, high concentrations of lactic acid bacteria (LAB) have been identified [4], suggesting that tocosh serves as a reservoir of functional microbial biodiversity.

LAB are recognized for synthesizing antimicrobial compounds that antagonize pathogens, thereby ensuring the microbiological safety of fermented foods [5]. However, the diversity and physiological behavior of these microorganisms are strongly influenced by ecological factors such as altitude [6,7], which can also facilitate the geographical authentication of traditional fermented products [8]. Elucidating this ecological and genomic diversity is fundamental for evaluating the quality, authenticity, and safety of fermented foods, as well as for bioprospecting new probiotic strains with optimized functional traits [7,9,10]. Given that fermented food are globally acknowledged as key modulators to gut microbiota in human health [11], traditional products like tocosh offer significant potential for the discovery of novel probiotics.

Beyond their ecological relevance, LAB play a crucial role in human health. A large-scale metagenomic analysis of 9445 human microbiomes revealed that fermented foods constitute a significant source of LAB for the gut microbiota, with prevalence and abundance varying according to age, lifestyle, and geography [12]. This highlights the importance of food-borne LAB such as *Streptococcus thermophilus* and *Lactococcus lactis* in shaping the gut ecosystem and modulating the gut–liver–brain axis, a key regulatory network linked to metabolic and neurodegenerative diseases [13]. LAB synthesize a diverse array of bioactive metabolites, including γ-aminobutyric acid (GABA), phenylethylamine, bioactive peptides, short-chain fatty acids, conjugated linoleic acid, selenometabolites, and several vitamins [14]. Among these, riboflavin and folate are of particular interest due to their nutritional and biotechnological relevance [15,16]. Such metabolic versatility underpins the potential of LAB as natural biofactories for the design of functional foods and nutraceuticals. Whole genome sequencing (WGS) has recently emerged as a powerful approach to overcome the limitations of traditional in vitro assays for assessing probiotic properties. WGS enables strain-level differentiation and comprehensive risk assessment within the framework of food safety [17]. Through next-generation sequencing (NGS), probiogenomic studies can predict genes related to virulence, bacteriocin production, mobile genetic elements, antimicrobial and anti-inflammatory biomolecules, and antibiotic resistance determinants [18]. This genome-scale approach facilitates the identification of safe and functionally potent strains for biotechnological applications, aligning with international guidelines for probiotic evaluation [19].

Previous research has explored LAB diversity in tocosh using high-throughput sequencing (HTS) and culture-dependent methods, with species identification based on 16S rRNA sequencing [20]. Similarly, LAB communities in other traditional fermented products like chicha have been studied using ISR-PCR, RAPD-PCR, and species-specific PCR [21]. Nevertheless, tocosh-associated LAB remain poorly characterized at the genomic level. Although recent NGS-based studies have elucidated the complete genomes of LAB from fermented foods like sauerkraut [22] and chicha de jora [23], comparative genomic analyses of LAB isolated from tocosh at different altitudes have not been reported to date. Furthermore, the recent taxonomic reclassification of the *Lactobacillus* genus into 25 distinct genera underscores the need for updated genomic evaluations that accurately capture strain diversity and evolutionary adaptation [24,25].

Consequently, this study aims to provide a comprehensive genomic characterization of LAB isolated from tocosh produced at three distinct altitudes in the Peruvian Andes. Specifically, sequencing quality, genome assembly and annotation, taxonomic identification, and functional profiling were evaluated to elucidate their probiotic potential and genetic safety.

## 2. Results

### 2.1. Genome Assembly and Annotation

**Figure 1:**
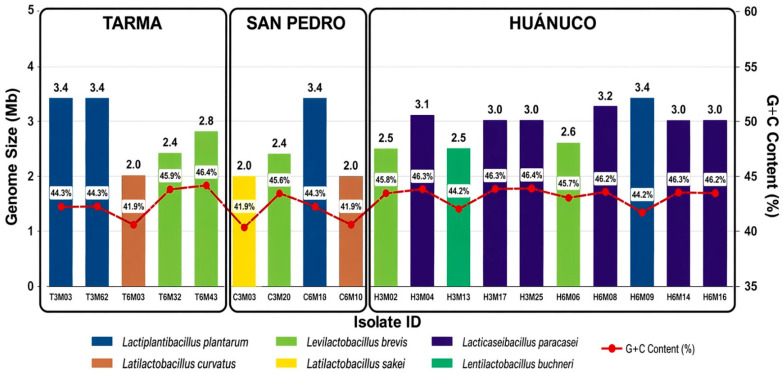
Comparative genomic architecture and G + C content of lactic acid bacteria (LAB) isolated from tocosh. The bar chart represents the genome size (Mb, left *y*-axis) of the 19 isolates categorized by species and sampling sites (Tarma, San Pedro de Cajas and Huanuco), color coded according to their taxonomic assignment (*L. brevis*, *L. buchneri*, *L. paracasei*, *L. plantarum*, *L. curvatus*, and *L. sakei*). The red dashed line and the secondary right *y*-axis are used to denote the G + C content (%) with specific values labeled within each bar. The values were derived from de novo assemblies generated with Unicycler and annotated with Bakta.

To characterize the genomic features of the isolates, whole genome sequencing (WGS) and subsequent genome annotation were performed on the 19 lactic acid bacteria isolated from tocosh, yielding high-quality draft genomes. Six distinct species were identified through taxonomic assignment: *Lactiplantibacillus plantarum* (UNCP-T3M03, UNCP-T3M62, UNCP-C6M04, UNCP-H6M09); *Latilactobacillus curvatus* (UNCP-T6M03 and UNCP-C6M10); *Levilactobacillus brevis* (UNCP-T6M32, UNCP-T6M43, UNCP-C3M20, UNCP-H3M02, UNCP-H6M06); *Latilactobacillus sakei* (UNCP-C3M03); *Lacticaseibacillus paracasei* (UNCP-H3M04, UNCP-H3M17, UNCP-H3M25, UNCP-H6M08, UNCP-H6M14, UNCP-H6M16); and *Lentilactobacillus buchneri* (UNCP-H3M13) (Figure 1).

As shown in Table 1, analysis of species distribution revealed that *L. plantarum* and *L. brevis* were present across all three sampling locations, suggesting robust adaptation to varying altitudinal gradients. *L. curvatus* and *L. sakei* were identified exclusively in samples from the highest altitudes. In contrast, *L. paracasei* and *L. buchneri* were identified exclusively in samples from Huanuco, the site at the lowest altitude (2992 m.a.s.l.).

Regarding functional machinery, the annotation pipeline identified a robust translation apparatus across all genomes. As demonstrated in Table 1, the number of tRNA genes ranged from 36 in *L. paracasei* H6M16 to 73 in *L. plantarum* H6M09, reflecting a diverse capacity for protein synthesis and environmental adaptation. Furthermore, the presence of CRISPR arrays were detected in most of the isolates (16 out of 19), indicating active adaptive immunity systems against phage attacks within the tocosh fermentation niche.

As illustrated in Figure 1, isolates belonging to *L. plantarum* and *L. paracasei* demonstrate the largest and the most stable genome size, in comparison to the other *Lactilactobacillus* species.

### 2.2. Functional Characterization and Probiotic Potential

**Figure 2:**
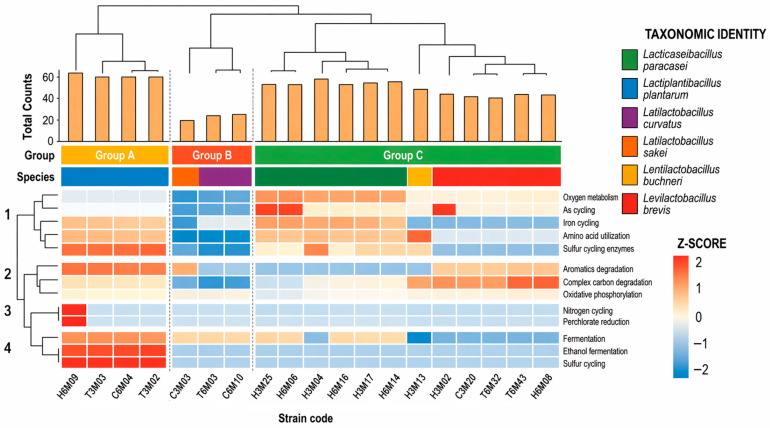
Comparative analysis of the functional landscape of tocosh-associated LAB isolates. Clustered heatmap illustrating the standardized abundance (Z-scores) of metabolic pathways across 19 genomes. The heatmap was generated using hierarchical clustering based on Ward’s method and Euclidean distance. The top dendrogram identifies three functional cohorts: Group A (*L. plantarum*), Group B (*L. curvatus* and *L. sakei*), and Group C (*L. paracasei*, *L. buchneri* and *L. brevis*). Bar plots represent the total functional counts per isolate. Colored strips indicate cluster membership and taxonomic identity. The “tile” effect highlights the specific contribution of each isolate to nitrogen, sulfur, and carbon cycling. Visualization was generated by R software version 4.5.2 using the *ComplexHeatmap* package.

To evaluate the metabolic capabilities of the tocosh-derived isolates, functional profiling was performed based on standardized gene counts. Hierarchical clustering analysis of the 19 validated genomes revealed three distinct functional groups (A, B, and C), driven by specific metabolic priorities (Figure 2).

Group A, exclusively composed of *L. plantarum* isolates, exhibited the highest functional counts (~60 genes) and a robust metabolic profile. This group showed significantly high Z-scores in critical pathways for the fermented potato niche, including nitrogen cycling, perchlorate reduction, and iron cycling (Cluster 3 and 4). Conversely, Group B, composed of *L. curvatus* and *L. sakei,* presented the most reduced metabolic profile, with total functional counts dropping to approximately 20 genes. Group C, the most taxonomically diverse cluster (*L. paracasei*, *L. brevis*, and *L. buchneri*), demonstrated a balanced and moderate metabolic activity (total counts ~40–50 genes). This group was characterized by high activity in amino acid utilization, oxygen metabolism, and complex carbon degradation (Cluster 1 and 2), highlighting their contribution to the biochemical maturation and safety of traditional tocosh.

**Figure 3:**
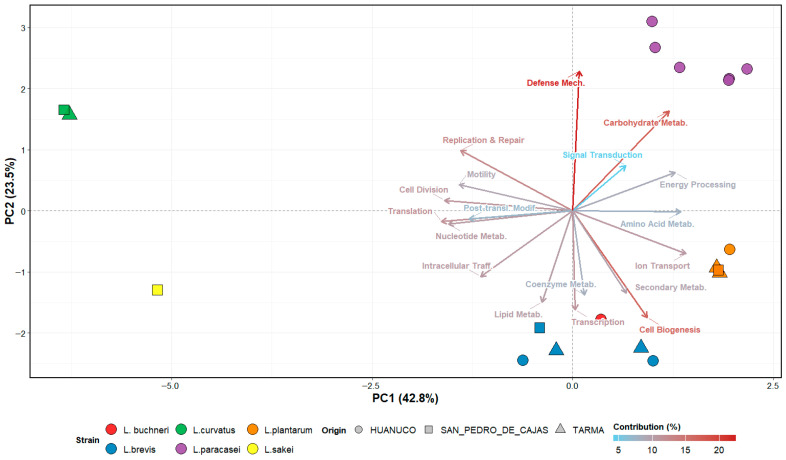
PCA of COG-based functional profiles of LAB isolated from tocosh. The biplot illustrates the distribution of 19 LAB genomes across the first two principal components (PC1 = 42.8%, PC2 = 23.5%). Points are colored by species and shaped by the origin of the samples (Huanuco, San Pedro de Cajas, and Tarma). Arrows represent the contributions of COG functional categories (loadings), with major drivers including defense mechanisms, carbohydrate metabolism, and ion transport. COGs were annotated using *METABOLIC* (incorporating KEGG, EggNOG, and GO databases), normalized by Z-score, and analyzed via PCA. This visualization highlights species-specific functional specialization, with *L. paracasei* displaying the greatest potential for defensive mechanisms.

Principal component analysis (PCA) further elucidated these functional divergences (Figure 3), explaining 66.3% of the total variance (PC1: 42.8%; PC2: 23.5%). The analysis revealed that separation along PC1 was predominantly driven by trade-offs between housekeeping functions and metabolic processing. Specifically, negative loadings were associated with translation (loading = −0.34) and cell division (loading = −0.33), whereas positive loadings were dominated by ion transport (loading = 0.29) and amino acid metabolism (loading = 0.28).

Notably, in the context of probiotic assessment, PC2 revealed a pronounced divergence based on defense mechanisms (loading = 0.47) and carbohydrate metabolism (loading = 0.34).

### 2.3. Relative Abundance of Carbohydrate-Active Enzymes (CAZy)

**Figure 4:**
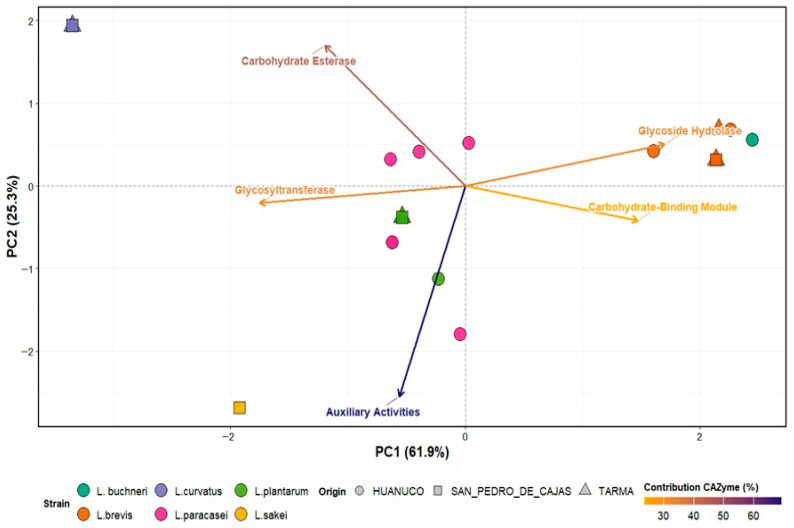
Principal component analysis (PCA) of CAZyme functional profiles in tocosh-derived LAB. The biplot illustrates the distribution of LAB genomes according to their CAZyme repertoires (PC1 = 61.9%, PC2 = 25.3%). The points represent isolates, colored by species and shaped by origin of the samples. Arrows indicate major CAZyme families (GH, GT, CE, CBM, AA), with the lengths proportional to their loadings (contributions). CAZymes were annotated using *dbCAN2*, normalized as Z-scores, and analyzed by PCA. The figure highlights the functional contrast between carbohydrate-degrading strains (GH/CBM-rich) and strains enriched in anabolic or oxidative functions (GT/AA-rich).

Considering the starch-dense composition of the tocosh matrix (Figure 4), the genomic repertoire of carbohydrate-active enzymes was evaluated to determine the substrate degradation potential of the isolates. Principal component analysis (PCA) of the CAZy profiles explained 87.2% of the total variance, revealing a distinct functional dichotomy between anabolic and catabolic capabilities. The primary axis (PC1, 61.9%) established a clear separation driven by the trade-off between glycosyltransferases (GT) and glycoside hydrolases (GH). As outlined in the loading analysis, GTs showed a strong negative contribution (loading = −0.56), correlating with isolates such as *L. curvatus* (purple triangle) and *L. sakei* (yellow square), positioned in the left quadrants. This finding suggests a genomic investment in the biosynthesis of exopolysaccharides or cell wall structures rather than in complex hydrolysis.

Conversely, GH (loading = 0.54) and carbohydrate binding modules (CBM) (loading = 0.47) were the main drivers of the positive PC1 axis. Isolates of *L. brevis* (orange symbols) and *L. buchneri* (teal circle) clustered strongly in this direction, indicating a specialized capacity for complex carbohydrate hydrolysis critical for potato starch breakdown.

The second component (PC2, 25.3%) was defined by carbohydrate esterases (CE) (loading = 0.54) versus auxiliary activities (AA) (loading = −0.81). *L. plantarum* isolates (green symbols) and *L. sakei* were associated with the negative PC2 axis, particularly with AA. This suggests a distinctive oxidative capacity (e.g., lytic polysaccharide monooxygenases) that may facilitate hydrolase access to recalcitrant starch granules in the *tocosh* environment.

**Figure 5:**
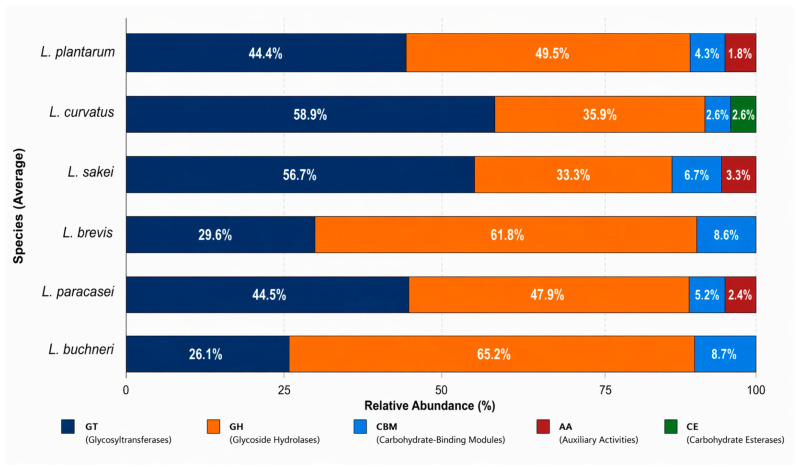
Relative abundance of CAZyme families across tocosh-associated LAB species. The stacked bars illustrate the percentage distribution of glycosyltransferases (GTs), glycoside hydrolases (GHs), carbohydrate binding modules (CBMs), auxiliary activities (AAs), and carbohydrate esterases (CEs)—for each identified LAB species. Data represent the mean relative abundance within each taxonomic group. CAZymes were annotated using *dbCAN2* and expressed as percentages of the total CAZyme content per genome.

To quantify the functional specialization observed in the PCA, the relative abundance of CAZy families was calculated for each species (Figure 5). The analysis confirmed a marked contrast in the allocation of genomic resources between hydrolytic and biosynthetic machinery. Glycoside hydrolases (GHs), responsible for the cleavage of glycosidic bonds in complex carbohydrates, constituted the dominant category in *L. buchneri* (65.2%) and *L. brevis* (61.8%). The high proportion of GHs, accompanied by a significant presence of CBM (8.7% in both species), underscores the specialized role of these species as primary degraders within the tocosh fermentation matrix, equipped to bind and hydrolyze starch granules efficiently.

Conversely, *L. curvatus* and *L. sakei* exhibited profiles dominated by GTs, reaching relative abundances of 59.0% and 56.7%, respectively. This predominance directs their metabolic energy towards biosynthesis of exopolysaccharides (EPS). Notably, *L. plantarum* and *L. paracasei* displayed the most balanced functional profiles, with a near-equitable distribution between catabolic (GH: 47.9–50.0%) and anabolic (GT: 44.4–44.5%) enzymes. This equilibrium reflects their ecological versatility, allowing them to switch between substrate scavenging and biomass production. Furthermore, *L. curvatus* strains were the only species associated with carbohydrate esterase (CE) (2.6%), potentially conferring unique capabilities for the deacetylation of hemicelluloses or other modified plant polysaccharides.

### 2.4. Pathogenicity and Safety Assessment

**Figure 6:**
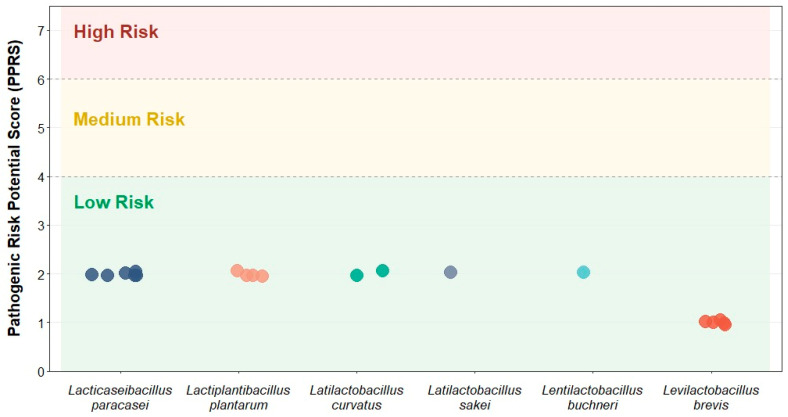
Pathogenic risk potential score (PPRS) of tocosh-derived LAB isolates. The pathogenic potential of the 19 isolates was evaluated using the pathogenic risk potential score (PPRS), which integrates data from the CARD (antibiotic resistance genes—ARGs), VFDB (Virulence Factors), and PHI-base (pathogen-host interactions) databases.

Ensuring the genomic safety of LAB is essential for their effective application within food systems. Genomic screening revealed the absence of transferable antibiotic resistance genes (ARGs) and virulence factors (VFs) across all isolates. Accordingly, PPRS values consistently range between 1.0 and 2.0, placing all strains well within the low-risk category (threshold < 4.0) (Figure 6).

These results confirm that the validated isolates possess an optimal safety profile, regardless of species-specific genomic variations, establishing them as suitable candidates for probiotic applications.

## 3. Discussion

### 3.1. Genomic Characteristics of Lactobacillus Species

The genomic characterization of tocosh-derived LAB revealed a distinct taxonomic distribution shaped by the high-altitude Andean environment. A striking observation in the validated cohort (19 strains) is the diversity in genome sizes, ranging from 1.96 Mb in *L. curvatus* to 3.38 Mb in *L. plantarum*. Such genomic heterogeneity, within the tocosh matrix, supports the concept of functional complementarity, where coexisting species occupy differentiated metabolic roles rather than direct competition [26,27].

*L. plantarum* strains demonstrated remarkable genomic stability across different geographical locations [28]. *L. brevis* aligns with its capability to thrive in spontaneously fermented vegetable products [29].

The detection of *L. sakei* and *L. curvatus* at high elevations is ecologically significant [30]. These species are typically associated with fermented meat rather than plant-based matrices [31]. Their presence in tocosh likely reflects specific adaptation to the cold, anaerobic conditions of the fermentation wells.

*L. paracasei* isolates showed genome sizes ranging from 3.01 to 3.22 Mb, exhibiting the highest G + C content in this cohort (46.2–46.4%) and were found at the lowest altitude. The genome sizes found here are similar to seven *L. paracasei* strains with a G + C content between 46.11% and 46.17% supporting their classification as nomadic lactobacilli [32].

There are no studies on differences in the size of lactic acid bacteria genomes due to altitude, but there are studies on differences due to different niches. The genome sizes of *L. plantarum* strains isolated from tocosh in this study ranged from 3.36 Mb at 4445 m.a.s.l. to 3.38 Mb at 2992 m.a.s.l., showing a very small difference. However, it is within the appropriate range from 2.61 Mb to 3.90 Mb through the analysis of 1240 *L. plantarum* strains [33]. These results indicate a stable coding capacity across the species at different altitudes, consistent with its metabolic versatility and adaptation to diverse niches, and a highly conserved genomic architecture [34].

In this research, the coding DNA sequence (CDSs) of *L. plantarum* ranged from 3187 at the highest altitudes to 3216 at the lowest altitudes. This pattern indicates that at higher altitudes, the genome size is slightly smaller, and the CDS is correspondingly lower. Notably, CDS in tocosh-derived *L. plantarun* strains exceeded those reported for the reference strain *L. plantarum* WCFS1 (3052 predicted protein-encoding genes) isolated from human Saliva [35].

The genome size of *L. brevis* strains isolated from tocosh were slightly larger as altitude decreases 2.44 Mb at 4445 m.a.s.l. < 2.59 Mb at 3882 m.a.s.l. < 2.61 Mb at 2992 m.a.s.l.; however, they are within the size range of strains from different origins (2.3–2.7 Mb) [36].

Consistently, *L. brevis* coding DNA sequences (CDSs), in this research, were 2416 at the highest altitude and 2575 at the lowest altitude, indicating a positive association between genome size and CDS. Comparative data show that *L. brevis* isolated from beer have an average of 2385 CDSs, whereas strains isolated from food, silage, animal intestines, and non-spoilage brewery environments have an average of 2311 CDSs [36]. Thus, tocosh-derived *L. brevis* strains exhibit relatively higher CDSs, suggesting an expanded coding potential.

The identification of *L. brevis* as a dominant species aligns with its capability to thrive in spontaneously fermented vegetable products [29]. Our findings suggest that strain-specific genomic plasticity is a primary adaptive strategy in tocosh.

The tocosh environment appears to favor a robust genomic repertoire for *L. plantarum*, which maintains a conserved size (~3.3 Mb), whereas *L. curvatus* isolates exhibited the shortest genome size (~1.9 Mb), consistent with niche specialization [34]. This dichotomy suggests that the tocosh ecosystem exerts dual evolutionary pressures: one selecting for metabolic versatility and another for efficiency through genome streamlining. The presence of CRISPR arrays in 16 out of 19 isolates further supports the hypothesis that these genomes function as active reservoirs of defense against high viral (phage) pressure in the fermentation wells [37].

### 3.2. Functional Characterization of Lactobacillus Species

As shown in Figure 3, *L. paracasei* isolates (purple circles) were strongly associated with the defense mechanisms vector, indicating a genetic repertoire equipped for persistence and competition, traits commonly linked to gut colonization and antagonist activity against pathogens [38]. Conversely, *L. curvatus* (green squares) and *L. sakei* (yellow squares) clustered negatively along PC1, showing a stronger association with replication and repair machinery which reflects their adaptation to environmental stress and genomic stability in dynamic fermentation systems [39]. *L. plantarum* isolates (orange symbols) clustered positively along PC1, aligning with high ion transport and secondary metabolite production, features associated with ecological versatility, stress tolerance and probiotic functionality [40,41], suggesting their ubiquitous nature and adaptative capacity of persisting in diverse niches [27]. Similarly *L. brevis* (light blue symbols) showed a positive clustering along PC1 with a stronger association to lipid metabolism and cell wall biogenesis, suggesting a role in membrane adaptation and environmental resilience, which may contribute to its functional performance in fermentation and gastrointestinal environments [42].

According to Figure 2, *L. plantarum* (group A) plays a primary role in mineral bioavailability and nutrient transformation during the tocosh production process. *L. curvatus* and *L. sakei* (group B) exhibited the most reduced metabolic profiles. *L. paracasei, L. brevis* and *L. buchneri* (group C), the most taxonomically diverse cluster, were characterized by high activity in amino acid utilization, oxygen metabolism and complex carbon degradation; these results highlight their contribution to biochemical maturation and safety traditional tocosh besides its diversity and biotechnological potential [20].

*L. paracasei* H3M25 and H6M06 and *Lactobacillus brevis* H3M02, isolated from the lowest altitude sites in this study, harbor a genetic repertoire associated with the arsenic cycle. Specifically, *arsC* (*trx*), which encodes a thioredoxin-dependent cytoplasmic arsenate reductase implicated in detoxification, was identified. Functionally, ArsC catalyzes the intracellular arsenate reduction. ArsC together with ArxA/AioA suggest a potential for integrated arsenic redox cycling, contributing to the maintenance of As(III)/As(V) gradients and coupling with other biogeochemical cycles [43]. These detoxification mechanisms of inorganic arsenic (iAs) have been reported in several *Lactobacillus* species [44].

Various *Lactobacillus* species, including *L. paracasei* BL23 [45] and *L. brevis* BL36 [46], have demonstrated biosorption capabilities and the ability to reduce As(III) toxicity, in both in vitro and in vivo models, by reducing intestinal absorption and activating antioxidant and immunomodulatory responses [47].Inorganic arsenic is more toxic than organic forms, and trivalent species (As(III), arsenite) are more toxic than pentavalent (As(V), arsenate) ones. It has been linked to the development of various types of cancer [48].

Tocosh is produced using water sourced from high-altitude mountain streams that flow continuously and from native potatoes. These potatoes have been reported to have low levels of total arsenic (TAs) and are unlikely to pose a significant health risk to consumers. In some cases, arsenic is predominantly localized in the potato skin, which can be removed by peeling, thereby eliminating most of the arsenic content [49]. Collectively, these findings suggest that tocosh represents a low-risk product in terms of arsenic contamination.

According to this research, *L. plantarum* genomes harbor genes associated with the “sulfur cycle” indicating a metabolic potential to participate in sulfur biogeochemical cycles, generally through oxidation-reduction pathways. In the case of tocosh, sulfur reduction could occur in the underwater sediment while oxidation does in the pond’s surface waters, creating a dynamic equilibrium driven by key functional genes. These include *dsrB* for sulfate reduction, *soxB* for sulfur oxidation, and *dsyB* for the biosynthesis of dimethylsulfonylpropionate (DMSP) [50].

Sulfur in the intestine originates from both dietary sources (cysteine, methionine, and sulfated compounds) and host-derived sulfated mucins. Consequently, *L. plantarum* may have bidirectional redox system with intestinal sulfur cycling, whereas microbial reduction generates H_2_S (sulfate → sulfite → H_2_S via sulfate-reducing bacteria and cysteine → H_2_S via desulfhydration) and host-mediated oxidation contributes to its detoxification (H_2_S → thiosulfate/tetrathionate via mitochondrial oxidation pathways). This host–microbiota co-evolved system tightly regulates H_2_S levels, where physiological concentrations exert beneficial effects, while excessive accumulation is associated with colonic pathology [51].

### 3.3. Carbohydrate-Active Enzymes (CAZy) of Lactobacillus Species

The predominance of glycosyl hydrolases in *L. buchneri* (~65.2%) and *L. brevis* (~61.8%), suggests a strong carbohydrate-degrading capacity that can be directly linked to their probiotic potential. Glycosyl hydrolases are key components of the CAZyme system, enabling the breakdown of complex polysaccharides and non-digestible oligosaccharides into fermentable sugars [52]. This metabolic capability supports the production of organic acids and other bioactive metabolites, which contribute to intestinal acidification and inhibition of competing microorganisms [53]. GHs may also play a role in the degradation of biofilms and exopolysaccharides of fungi and bacteria [54]. These species, especially *L. brevis*, exhibit heterofermentative metabolism, generating a broader range of metabolites, which may enhance their antagonistic activity against pathogens and modulate gut microbial dynamics [40].

*L. plantarum* strains, observed at different altitudes in this study, typically possess a high abundance of glycosyl hydrolases (GHs) and glycosyltransferases (GTs). These findings are consistent with previously reported GH and GT families in *L. plantarum* (GH1, GH13, GT2, GT4) which demonstrate its adaptability to diverse ecological niches and its probiotic functionality [28,41]. Furthermore, CAZyme-rich strains of *L. plantarum* have been associated with enhanced survival under gastrointestinal stress, antimicrobial activity, and host interaction, reinforcing their probiotic potential [40].

On the other hand, the predominance of GTs in *L. curvatus* (~59%) and *L. sakei* (~56.7%), highlights their strong biosynthetic capacity for exopolysaccharides (EPS) and surface glycoconjugates, which are key determinants of probiotic functionality. EPS produced by lactic acid bacteria have been widely associated with enhanced adhesion to intestinal mucosa, biofilm formation, and protection against environmental stresses such as low pH and bile salts, thereby improving bacterial survival in the gastrointestinal tract [55]. Furthermore, these biopolymers exhibit important bioactivities, including immunomodulatory effects, regulation of cytokine responses and enhancement of intestinal barrier function [56], as well as antioxidant and microbiota-modulating effects, contributing to host health [57].

The functional profiling via CAZymes analysis elucidated how these strains partition metabolic roles to deconstruct the potato matrix. The observed dichotomy between “degraders” (high GH/CBM content in *L. brevis* and *L. buchneri*) and “builders” (high GT content in *L. curvatus* and *L. sakei*) implies a synergistic community network. *L. brevis* and *L. buchneri* likely act as primary degraders, hydrolyzing raw starch granules to release fermentable sugars [52]. Interestingly, *L. plantarum* was associated with auxiliary activities (AAs), suggesting it may employ oxidative enzymes to disrupt the recalcitrant surface of raw potato starch, facilitating the action of hydrolases [58]. On the other hand, the enrichment of glycosyltransferases (GTs) in *L. curvatus* and *L. sakei* suggests that these species allocate metabolic resources towards anabolic processes like exopolysaccharide (EPS) biosynthesis. In high-altitude fermentation, EPS likely serves a cryoprotective function, preventing cell damage during freezing temperatures [59].

*L. plantarum, L. sakei* and *L. paracasei* were also associated with auxiliary activities including lytic polysaccharide monooxygenases (LPMOs) which contribute to the oxidative breakdown of complex biomass, thereby facilitating the action of hydrolases [60].

*L. curvatus* is the only species associated with carbohydrate esterases which hydrolyze ester bonds on carbohydrate backbones, removing ester modifications (like acetyl or methyl groups) from mono-, oligo-, and polysaccharides. These enzymes play a critical role in degrading complex biomass, such as plant cell walls, by enabling glycosyl hydrolases to access and break down the remaining sugar structures [61].

### 3.4. Safety Assessment of Lactobacillus Species

From a safety and probiotic perspective, the in-silico assessment indicates that traditional tocosh fermentation may function as a biological safety filter. The low PPRSs (≤2.0) and the complete absence of transferable antibiotic resistance genes (ARGs) and virulence factors (VFs) across all 19 genomes support the safety of these isolates for human consumption, consistent with the qualified presumption of safety (QPS) criteria [62].

A focused investigation of *Lactobacillus buchneri*, also identified in this study, revealed the absence of tetracycline resistance genes within the studied strains, highlighting the variability in antibiotic resistance profiles among probiotic species [63].

Recent evidence confirms that *Lactobacillus species* exhibit conserved intrinsic resistance to several antibiotic classes, particularly glycopeptides and aminoglycosides. Although resistance genes have been identified, they have not been associated with mobile genetic elements, thereby reducing the risk of dissemination and posing minimal safety concern in food and probiotic applications [64,65].

## 4. Materials and Methods

### 4.1. Study Area and Sample Collection

**Figure 7:**
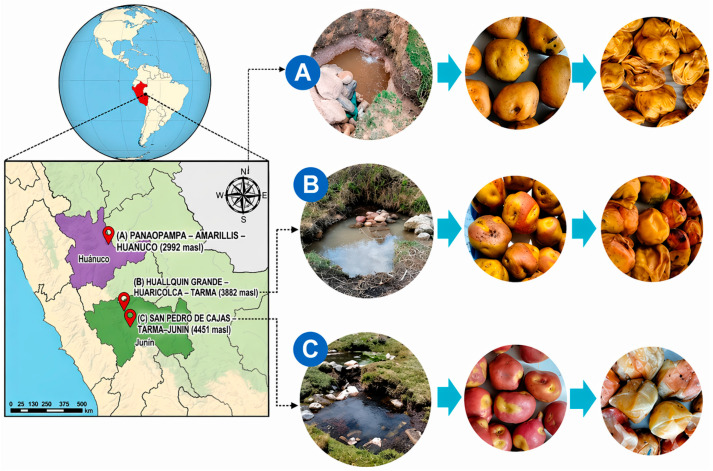
Geographical sampling locations and traditional tocosh fermentation workflow. Map of the study areas in the central Andes, illustrating the linking among the specific fermentation niches (natural springs), native potato varieties, and the resulting tocosh product across an altitudinal gradient.

Tocosh samples were prepared using native yellow potatoes cultivated in the studied sites. The production was carried out in traditional earthen wells located near natural springs at three specific high-altitude locations in the Peruvian Andes (Figure 7).

As shown in Figure 7, the sampling sites were: (A) Panaopampa (Amarillis, Huanuco) at 2992 m above sea level (m.a.s.l.); (B) Huallquin Grande (Huaricolca, Tarma) at 3882 m.a.s.l.; and (C) Caracuchan Mountain (San Pedro de Cajas, Junin) at 4451 m.a.s.l.

Detailed environmental parameters of each site are provided in Table 2. Potatoes were submerged in wells and fermented for two distinct periods: 3 months and 6 months. After the fermentation period, samples were collected aseptically, transferred to the laboratory under cold chain conditions, and peeled prior to analysis. The average weight of the tocosh samples was 21.5 ± 5.5 g after three months and 18.8 ± 3.5 g after six months across the three production sites.

### 4.2. Isolation of Lactic Acid Bacteria

Ten grams (10 g) of each tocosh sample were homogenized with 90 mL of 0.85% (*w*/*v*) sterile saline solution for 3 min using a Stomacher Bag Mixer 400 cc (Interscience, Saint Nom la Bretèche, France). Serial decimal dilutions were prepared and plated in triplicate onto MRS agar (CM0361B, Oxoid, Basingstoke, UK) supplemented with 0.1% cycloheximide (Sigma-Aldrich, Burlington, MA, USA) to inhibit fungal and yeast proliferation [66]. Plates were incubated anaerobically at 32 °C for 48 h. Colonies exhibiting distinct morphological characteristics were selected from plates containing 30–300 colonies. A total of 202 isolates were recovered (117 from Huallquin grande, 44 from San Pedro de Cajas, and 41 from Panaopampa). Isolates confirmed as Gram-positive, catalase-negative [67], and oxidase-negative (Bactident oxidase, 50 strips) were considered presumptive lactic acid bacteria (LAB) and stored at −70 °C in MRS broth supplemented with 20% (*v*/*v*) glycerol.

### 4.3. Genomic DNA Extraction and Genotypic Fingerprinting (Rep-PCR)

Genomic DNA was extracted using the GeneJET Genomic DNA Purification Kit (Thermo Scientific, Waltham, MA, USA) following the manufacturer’s instructions. DNA quality and concentration were assessed by spectrophotometry (Nanodrop One, Thermo Scientific USA); samples showed concentrations ranging from 30.63 to 56.58 ng/µL, A260/A280 ratios from 1.73 to 2.07, A260/230 ratios from 1.85 to 2.90. To select representative strains for sequencing, genotypic characterization was performed by repetitive sequence-based PCR (rep-PCR) using the (GTG) 5 primer [68].

The PCR amplification was carried out using the Gotaq^®^ G2 Flexi DNA polymerase kit—(Promega, Madison, WI, USA).

The final volume of the reaction was 20 µL, contained 4 μL of 5 X buffer, 0.4 μL of 10 mM dNTP, 0.1 μL of Taq polymerase (5 U/μL), 1 μL of template DNA (~50 ng), 1.6 μL of primer (5′-GTGGTGGTGTGTGG-3′), and 3.2 μL of MgCl_2_ (25 mM); therefore, the final concentration of the components was Buffer green 1X, MgCl_2_ (4 mM), dNTPs (0.2 mM), Taq polymerase (0.025 U/µL).

Amplification was carried out in a Biometra T One 96G thermocycler (Analytic Jena, Jena, Germany) with the following conditions: an initial denaturation at 94 °C for 5 min; 30-cycle reaction: 1 min denaturation at 94 °C, 1 min annealing at 40 °C, 8 min extension at 65 °C and final extension at 65 °C for 10 min.

PCR products were separated by electrophoresis on 1.5% (*w*/*v*) agarose gels pre-stained with SafeView Classic G108 (Applied Biological Materials Inc., Richmond, BC, Canada) at 65 V for 2 h at room temperature in 1X TAE buffer solution. Gel images were recorded with a BIO-RAD photo documentation device (GelDoc Go Imaging System—Bio-Rad Laboratories, Hercules, CA, USA) and stored as TIFF files which were analyzed using GelJ v2.0 software [69]. Cluster analysis was performed using the unweighted pair group method with arithmetic mean (UPGMA) based on Pearson’s correlation coefficients. Isolates, with ≥95% similarity, were considered the same biotype. Representative strains from unique clusters (<95% similarity) were selected for whole genome sequencing (WGS).

### 4.4. Whole Genome Sequencing

Nineteen distinct isolates were subjected to whole genome sequencing (WGS) using the Illumina MiSeq platform (Illumina, San Diego, CA, USA) for v2/v3 reagent kits. Genomic DNA was quantified using the Qubit dsDNA Broad Range Assay Kit on a Qubit 4 Fluorometer (Invitrogen, Thermo Fisher Scientific, Waltham, MA, USA). Sequencing libraries were prepared using the Illumina DNA Prep (M) Tagmentation kit according to the manufacturer’s instructions. This procedure involved the tagmentation of genomic DNA with bead-linked transposomes (BLTs), followed by post-tagmentation cleanup and library amplification using the enhancement PCR Mix (EPM) and IDT/Illumina UD Indexes (Set A).

The final library pool (20 pM) was spiked with 1% PhiX control and loaded onto an Illumina MiSeq Reagent Kit v3 (600-cycle) cartridge. Sequencing was performed in paired-end mode (2 × 151 bp). Raw reads were generated in FASTQ format after approximately 36 h of runtime.

### 4.5. Bioinformatic Analyses

#### 4.5.1. Sequencing Quality Control and Pre-Processing

The sequencing run yielded an average depth exceeding 300,000 reads per genome, providing sufficient coverage for the de novo assembly of LAB genomes (estimated size ≈ 2–4 Mb).

Raw data quality was assessed using FastQC v0.12.1 [70]. All datasets exhibited mean Phred scores (Q-scores) > 30 across the read length, corresponding to a base-call accuracy of ≥99.9%. This high-quality baseline minimized the risk of error propagation in downstream analyses. Subsequently, raw reads were processed using FastP v1.3.3 [71] to perform adapter trimming, filter low-quality bases, and remove duplicates. The resulting “clean reads” retained the original insert size distribution while being free of technical artifacts, thereby optimizing the dataset for precise open reading frame (ORF) prediction and annotation. Quality control metrics for all samples were aggregated and summarized using MultiQC v1.28 [72]. Only datasets passing these stringent quality criteria (average Q30 and successful adapter removal) proceeded to the assembly stage.

#### 4.5.2. Genomic Assembly and Annotation

Genomes were assembled de novo using Unicycler v0.5.1 with default parameters [73]. Assembly integrity and quality metrics (Table S1), including N50, contig counts, contamination and total genome size were assessed using QUAST v5.2.0 [74]. Following assembly validation, structural and functional annotation was performed using Bakta v1.11.0 [75] to identify coding sequences (CDSs), transfer RNAs (tRNAs), ribosomal RNAs (rRNAs), and CRISPR arrays.

#### 4.5.3. Taxonomic Classification

Taxonomic assignment was performed using GTDB-Tk tk v2.4.1 [76] based on the Genome Taxonomy Database (GTDB) standards. Species-level identification was further confirmed via the Type (Strain) Genome Server (TYGS) [77], referencing the LPSN database to ensure up-to-date nomenclature. This comprehensive bioinformatic pipeline integrated raw data processing, assembly, functional annotation and safety assessment to characterize the isolates.

### 4.6. Probiotic and Functional Profiling

Metabolic reconstruction was performed using the METABOLIC version 4.0 software [78], incorporating KEGG, EggNOG, and Gene Ontology (GO) annotations. The identification of carbohydrate-active enzymes (CAZymes) was conducted by searching against the dbCAN2 database. To ensure that the observed differences in CAZyme abundance were not biased by genome size variation, all CAZyme counts were normalized to the total number of protein-coding sequences (CDSs) per genome using the ProbioMinServer version 1.0 [79]. This approach enables rigorous comparison of metabolic investment in substrate degradation across the diverse taxonomic groups identified.

Probiotic safety was also evaluated by using the ProbioMinServer which screens for antibiotic resistance genes (ARGs) referencing the CARD database, and virulence factors (VFs) through VFDB and PHI-base. Probiotic potential risk score (PPRS) was calculated according to [80] classifying strains as low risk (≤4), medium risk (4–6), or high risk (≥6).

### 4.7. Statistical Analysis

Data processing, multivariate analysis, and visualization were performed using Python (v3.x) within the Jupyter Notebook environment version 7.4.5. Raw datasets were organized and curated using the pandas library. To evaluate genomic and functional dissimilarities, principal component analysis (PCA) was conducted using the scikit-learn library. To ensure mathematical consistency in the PCA projection, data were normalized using the StandardScaler module; this step scales variables to unit variance without altering their relative distribution. The resulting principal components (PCs) were visualized using matplotlib and seaborn, with data points labeled and optimized for readability using the adjustText algorithm. Hierarchical clustering analysis (HCA) was performed to group strains based on their metabolic similarities. A clustered heatmap was generated using seaborn, employing Euclidean distance as the similarity metric and Ward’s minimum variance method for linkage. The complementary cluster analysis was performed in the R statistical software version 4.5.2 by using the ComplexHeatmap package.

## 5. Conclusions

The probiogenomic characterization of lactic acid bacteria (LAB) isolated from tocosh indicates that the high-altitude Andean environment and starch-rich potato matrix drive the assembly of a specialized microbial architecture. Strains of *L. curvatus* and *L. sakei* from higher altitudes exhibit enrichment in replication and repair systems, indicating adaptation to environmental stress, alongside increased glycosyltransferase activity associated with exopolysaccharide biosynthesis, biofilm formation, and gastrointestinal resilience. In contrast, *L. paracasei* and *L. buchneri* from lower altitudes are associated with defense mechanisms and a predominance of glycosyl hydrolases, reflecting strong carbohydrate-degrading capacity and antagonistic activity against pathogens. *L. plantarum* and *L. brevis*, distributed across all altitudes, display metabolic versatility related to amino acid utilization, secondary metabolism, and cell biogenesis, coupled with combined glycosyl hydrolase and glycosyltransferase repertoires supporting both polysaccharide degradation and protective exopolysaccharide production. The absence of transferable antibiotic resistance genes and virulence factors supports a high safety profile consistent with QPS status. These in silico findings reveal significant probiotic potential and identify candidate strains for starter cultures; however, further experimental validation is required to confirm these genomic predictions. Overall, this research highlights these isolates as valuable biotechnological resources for safe, scalable applications.

## Figures and Tables

**Table 1:** Genomic characteristics and taxonomic identification of tocosh-derived LAB isolates.

Origin/ Altitude	Strain	Genome Size (bp)	Contigs	CDS	rRNA	tRNA	oriC	sORF	CRISPR	G + C (%)	Taxonomy GTDB-Tk	Taxonomy TYGS	NCBI Accession
San Pedro de Cajas—Tarma Junín 4451 m.a.s.l.	UNCP-C3M03	2,007,050	40	1998	2	54	1	9	0	41.0	*Lactobacillus sakei*	*Latilactobacillus* *sakei*	JBSVYN000000000
	UNCP-C3M20	2,444,486	65	2416	3	59	1	5	1	45.8	*Lactobacillus brevis*	*Levilactobacillus brevis*	JBSVYO000000000
	UNCP-C6M04	3,357,411	41	3187	3	71	2	1	1	44.3	*Lactobacillus plantarum*	*Lactiplantibacillus plantarum*	JBSVYP000000000
	UNCP-C6M10	1,963,444	77	1974	3	51	2	2	1	41.9	*Lactobacillus curvatus*	*Latilactobacillus curvatus*	JBSVYQ000000000
Huallquin grande— Huaricolca-Tarma 3882 m.a.s.l.	UNCP-T3M03	3,356,634	39	3185	3	69	2	1	1	44.3	*Lactobacillus plantarum*	*Lactiplantibacillus plantarum*	JBRERV000000000
	UNCP-T3M62	3,357,314	40	3188	3	71	2	1	1	44.3	*Lactobacillus plantarum*	*Lactiplantibacillus plantarum*	JBSVYJ000000000
	UNCP-T6M03	1,964,679	72	1974	4	51	2	2	2	41.9	*Lactobacillus curvatus*	*Latilactobacillus curvatus*	JBSVYK000000000
	UNCP-T6M32	2,410,130	61	2370	3	59	1	5	1	45.9	*Lactobacillus brevis*	*Levilactobacillus brevis*	JBSVYL000000000
	UNCP-T6M43	2,770,502	44	2774	3	67	2	1	1	45.4	*Lactobacillus brevis*	*Levilactobacillus brevis*	JBSVYM000000000
Panaopampa—Amarillis Huanuco 2992 m.a.s.l.	UNCP-H3M02	2,576,964	110	2579	2	61	2	2	1	45.6	*Lactobacillus brevis*	*Levilactobacillus brevis*	JBSVYR000000000
	UNCP-H3M04	3,105,671	108	2988	3	47	2	3	0	46.3	*Lactobacillus paracasei*	*Lacticaseibacillus paracasei*	JBSVYS000000000
	UNCP-H3M13	2,509,738	87	2401	3	56	2	4	1	44.2	*Lactobacillus buchneri*	*Lentilactobacillus buchneri*	JBSVYT000000000
	UNCP-H3M17	3,017,614	97	2833	4	48	3	3	1	46.3	*Lactobacillus paracasei*	*Lacticaseibacillus paracasei*	JBSVYU000000000
	UNCP-H3M25	3,042,223	139	2868	2	48	2	2	2	46.4	*Lactobacillus paracasei*	*Lacticaseibacillus paracasei*	JBSVYV000000000
	UNCP-H6M06	2,612,030	13	2571	3	56	1	2	1	45.7	*Lactobacillus brevis*	*Levilactobacillus brevis*	JBSVYW000000000
	UNCP-H6M08	3,223,644	258	3100	2	47	2	3	1	46.2	*Lactobacillus paracasei*	*Lacticaseibacillus paracasei*	JBSVYX000000000
	UNCP-H6M09	3,375,953	54	3216	2	73	2	1	0	44.3	*Lactobacillus plantarum*	*Lactiplantibacillus plantarum*	JBSVYY000000000
	UNCP-H6M14	3,010,899	94	2830	3	39	3	3	1	46.3	*Lactobacillus paracasei*	*Lacticaseibacillus paracasei*	JBSVYZ000000000
	UNCP-H6M16	3,022,862	88	2883	2	36	2	2	2	46.2	*Lactobacillus paracasei*	*Lacticaseibacillus paracasei*	JBSVZA000000000

**Table 2:** Average environmental conditions of the Tocosh production sites.

Environmental Conditions	Panaopampa Huanuco (H)	Huallquin Grande Tarma (T)	San Pedro de Cajas Tarma (C)
Political region	Huanuco	Junin	Junin
Altitude (m.a.s.l.)	2992	3882	4451
Latitude	9.989268 S	11.505310 S	11.257133 S
Longitude	76.188900 W	75.657620 W	75.865754 W
Atmospheric Pressure (hPa)	1019.6	1022.5	1018.2
Temperature (°C)	18.4	13.4	8.9
Relative Humidity (%)	88.1	57.3	82.7
Water Temp. of Wells (°C)	13.0	13.0	11.0

## Data Availability

The genomic data generated and associated information used in this research are deposited in the NCBI (https://www.ncbi.nlm.nih.gov/ (accessed on 10 May 2026)), NCBI accession on Table 1.

## Supplementary Materials

The following supporting information can be downloaded at: https://www.mdpi.com/article/10.3390/ijms27093981/s1.

## Abbreviations

The following abbreviations are used in this manuscript:

LAB	Lactic Acid Bacteria
WGS	Whole Genome Sequencing
NGS	Next-Generation Sequencing
m.a.s.l.	Meters above sea level
CDS	Coding DNA Sequences
CAZymes	Carbohydrate-Active Enzymes
GH	Glycoside Hydrolases
GT	Glycosyltransferases
CE	Carbohydrate Esterases
AA	Auxiliary Activities
CBM	Carbohydrate Binding Modules
EPS	Exopolysaccharides
ARGs	Antibiotic Resistance Genes
VFs	Virulence Factors
QPS	Qualified Presumption of Safety
PPRS	Probiotic Potential Risk Score
GTDB	Genome Taxonomy Database
TYGS	Type (Strain) Genome Server
LPSN	List of Prokaryotic Names with Position in Nomenclature
PCA	Principal Component Analysis
HCA	Hierarchical Clustering Analysis
UPGMA	Unweighted Pair Group Method with Arithmetic Mean
GABA	Gamma-aminobutyric acid

## References

[1] Yépez A. (2017). Biopreservation Potential of Lactic Acid Bacteria from Andean Fermented Food of Vegetal Origin. Food Control.

[2] Behera S.S., Kumar A., Ray R.C., Paramithiotis S., de Carvalho Azevedo V.A., Montet D. (2022). Chapter 4—Biotransformation of Root and Tuber Crops by Lactic Acid Bacteria into Value-Added Bio-Commodities. Lactic Acid Bacteria in Food Biotechnology.

[3] Núñez-González F.A., Millones-Gómez P. (2023). Tocosh: Penicilina natural de los andes y sus beneficios en la salud general. Med. Natur..

[4] Ramos E.R., Santos R.A., Velázquez E., Velezmoro C.E., Zúñiga D.E. (2018). Genetic Diversity and Antimicrobial Activity of Lactic Acid Bacteria in the Preparation of Traditional Fermented Potato Product ‘Tunta’. World J. Microbiol. Biotechnol..

[5] Agriopoulou S., Stamatelopoulou E., Sachadyn-Król M., Varzakas T. (2020). Lactic Acid Bacteria as Antibacterial Agents to Extend the Shelf Life of Fresh and Minimally Processed Fruits and Vegetables: Quality and Safety Aspects. Microorganisms.

[6] Hunduma T., Ashenafi M. (2011). Effect of Altitude on Microbial Succession during Traditional Enset (Ensete Ventricosum) Fermentation. Int. J. Food Nutr. Public Health.

[7] Koirala R., Ricci G., Taverniti V., Ferrario C., Malla R., Shrestha S., Fortina M.G., Guglielmetti S. (2014). Isolation and Molecular Characterization of Lactobacilli from Traditional Fermented Dahi Produced at Different Altitudes in Nepal. Dairy Sci. Technol..

[8] Bonizzi I., Buffoni J.N., Feligini M., Enne G. (2009). Investigating the Relationship between Raw Milk Bacterial Composition, as Described by Intergenic Transcribed Spacer-PCR Fingerprinting, and Pasture Altitude: Raw Milk Microflora and Altitude. J. Appl. Microbiol..

[9] Chourasia R., Chiring Phukon L., Minhajul Abedin M., Sahoo D., Kumar Rai A. (2022). Production and Characterization of Bioactive Peptides in Novel Functional Soybean *Chhurpi* Produced Using *Lactobacillus delbrueckii* WS4. Food Chem..

[10] Kulyar M.F., Mo Q., Nawaz S., Li J. (2025). High Altitude Microbiome: Insight into Yak Gut Microbiota and Its Nutritional and Functional Involvement for Food Systems. Trends Food Sci. Technol..

[11] Marco M.L., Heeney D., Binda S., Cifelli C.J., Cotter P.D., Foligné B., Gänzle M., Kort R., Pasin G., Pihlanto A. (2017). Health Benefits of Fermented Foods: Microbiota and Beyond. Curr. Opin. Biotechnol..

[12] Pasolli E., De Filippis F., Mauriello I.E., Cumbo F., Walsh A.M., Leech J., Cotter P.D., Segata N., Ercolini D. (2020). Large-Scale Genome-Wide Analysis Links Lactic Acid Bacteria from Food with the Gut Microbiome. Nat. Commun..

[13] Yan M., Man S., Sun B., Ma L., Guo L., Huang L., Gao W. (2023). Gut Liver Brain Axis in Diseases: The Implications for Therapeutic Interventions. Signal Transduct. Target. Ther..

[14] Pessione E. (2012). Lactic Acid Bacteria Contribution to Gut Microbiota Complexity: Lights and Shadows. Front. Cell. Inf. Microbiol..

[15] Mosso A.L., Jimenez M.E., Vignolo G., LeBlanc J.G., Samman N.C. (2018). Increasing the Folate Content of Tuber Based Foods Using Potentially Probiotic Lactic Acid Bacteria. Food Res. Int..

[16] Santos-Mendoza R., Ramos-Vásquez E., Iris Zavaleta A., Zúñiga-Dávila D., Velezmoro-Sánchez C. (2018). Bacterias Ácido Lácticas Productoras de Riboflavina Aisladas Del Proceso de Elaboración de La “Tunta”. Ecol. Apl..

[17] Peng X., Ed-Dra A., Yue M. (2023). Whole Genome Sequencing for the Risk Assessment of Probiotic Lactic Acid Bacteria. Crit. Rev. Food Sci. Nutr..

[18] Dos Santos T.G., Dos Santos K.A.G., De Oliveira E.J.F., Brenig B., Paulo E.M., Marques P.H., Cardoso V.N., Aburjaile F.F., Soares S.C., Da Silva W.M. (2025). Genomic Scale Analysis Foresees Enteroprotective and Butyrogenic Properties in Brazilian Isolates of *Lactiplantibacillus plantarum*. Probiot. Antimicrob. Proteins.

[19] FAO/WHO (2006). Health and nutritional properties and guidelines for evaluation. Probiotics in Food.

[20] Jiménez E., Yépez A., Pérez-Cataluña A., Ramos Vásquez E., Zúñiga Dávila D., Vignolo G., Aznar R. (2018). Exploring Diversity and Biotechnological Potential of Lactic Acid Bacteria from Tocosh—Traditional Peruvian Fermented Potatoes—By High Throughput Sequencing (HTS) and Culturing. LWT.

[21] Elizaquível P., Pérez-Cataluña A., Yépez A., Aristimuño C., Jiménez E., Cocconcelli P.S., Vignolo G., Aznar R. (2015). Pyrosequencing vs. Culture-Dependent Approaches to Analyze Lactic Acid Bacteria Associated to Chicha, a Traditional Maize-Based Fermented Beverage from Northwestern Argentina. Int. J. Food Microbiol..

[22] Karaseva O., Ozhegov G., Khusnutdinova D., Siniagina M., Anisimova E., Akhatova F., Fakhrullin R., Yarullina D. (2023). Whole Genome Sequencing of the Novel Probiotic Strain *Lactiplantibacillus plantarum* FCa3L. Microorganisms.

[23] Rodrigo-Torres L., Yépez A., Aznar R., Arahal D.R. (2019). Genomic Insights into Five Strains of *Lactobacillus plantarum* with Biotechnological Potential Isolated from Chicha, a Traditional Maize-Based Fermented Beverage from Northwestern Argentina. Front. Microbiol..

[24] Salvetti E., Torriani S., Felis G.E. (2012). The Genus Lactobacillus: A Taxonomic Update. Probiot. Antimicrob. Proteins.

[25] Zheng J., Wittouck S., Salvetti E., Franz C.M.A.P., Harris H.M.B., Mattarelli P., O’Toole P.W., Pot B., Vandamme P., Walter J. (2020). A Taxonomic Note on the Genus Lactobacillus: Description of 23 Novel Genera, Emended Description of the Genus Lactobacillus Beijerinck 1901, and Union of Lactobacillaceae and Leuconostocaceae. Int. J. Syst. Evol. Microbiol..

[26] Louca S., Polz M.F., Mazel F., Albright M.B.N., Huber J.A., O’Connor M.I., Ackermann M., Hahn A.S., Srivastava D.S., Crowe S.A. (2018). Function and Functional Redundancy in Microbial Systems. Nat. Ecol. Evol..

[27] Martino M.E., Bayjanov J.R., Caffrey B.E., Wels M., Joncour P., Hughes S., Gillet B., Kleerebezem M., Van Hijum S.A.F.T., Leulier F. (2016). Nomadic Lifestyle of *Lactobacillus plantarum* Revealed by Comparative Genomics of 54 Strains Isolated from Different Habitats. Environ. Microbiol..

[28] Li R., Bi C. (2025). Comparative Genomic Analysis of *Lactiplantibacillus plantarum*: Insights into Its Genetic Diversity, Metabolic Function, and Antibiotic Resistance. Genes.

[29] Gänzle M.G. (2015). Lactic Metabolism Revisited: Metabolism of Lactic Acid Bacteria in Food Fermentations and Food Spoilage. Curr. Opin. Food Sci..

[30] Park S., Son S., Park M.A., Kim D.-H., Kim Y. (2024). Complete Genome Sequence of Latilactobacillus Curvatus CACC879 and Its Functional Probiotic Properties. J. Anim. Sci. Technol..

[31] Zagorec M., Champomier-Vergès M.-C. (2017). Lactobacillus Sakei: A Starter for Sausage Fermentation, a Protective Culture for Meat Products. Microorganisms.

[32] Sornsenee P., Surachat K., Kang D.-K., Mendoza R., Romyasamit C. (2024). Probiotic Insights from the Genomic Exploration of *Lacticaseibacillus paracasei* Strains Isolated from Fermented Palm Sap. Foods.

[33] Wonglapsuwan M., Ninrat T., Chaichana N., Dechathai T., Suwannasin S., Singkhamanan K., Pomwised R., Surachat K. (2025). Global Genomic Landscapes of *Lactiplantibacillus plantarum*: Universal GABA Biosynthetic Capacity with Strain-Level Functional Diversity. Life.

[34] Makarova K., Slesarev A., Wolf Y., Sorokin A., Mirkin B., Koonin E., Pavlov A., Pavlova N., Karamychev V., Polouchine N. (2006). Comparative Genomics of the Lactic Acid Bacteria. Proc. Natl. Acad. Sci. USA.

[35] Kleerebezem M., Boekhorst J., Van Kranenburg R., Molenaar D., Kuipers O.P., Leer R., Tarchini R., Peters S.A., Sandbrink H.M., Fiers M.W.E.J. (2003). Complete Genome Sequence of *Lactobacillus plantarum* WCFS1. Proc. Natl. Acad. Sci. USA.

[36] Feyereisen M., Mahony J., Kelleher P., Roberts R.J., O’Sullivan T., Geertman J.-M.A., Van Sinderen D. (2019). Comparative Genome Analysis of the *Lactobacillus brevis* Species. BMC Genom..

[37] Barrangou R., Horvath P. (2017). A Decade of Discovery: CRISPR Functions and Applications. Nat. Microbiol..

[38] Dishan A., Gönülalan Z. (2025). *Lacticaseibacillus paracasei* AD22 Stress Response in Brined White Cheese Matrix: In Vitro Probiotic Profiles and Molecular Characterization. Probiot. Antimicrob. Proteins.

[39] Li K., Liu J., Zeng Z., Kulyar M.F.-A., Wang Y., Li A., Bhutta Z.A., Aqib A.I., Shahzad M., Li J. (2020). The Complete Genome of Probiotic *Lactobacillus sakei* Derived from Plateau Yak Feces. Genes.

[40] Filannino P., Di Cagno R., Gobbetti M. (2018). Metabolic and Functional Paths of Lactic Acid Bacteria in Plant Foods: Get out of the Labyrinth. Curr. Opin. Biotechnol..

[41] Gizachew S., Engidawork E. (2024). Genomic Characterization of *Lactiplantibacillus plantarum* Strains: Potential Probiotics from Ethiopian Traditional Fermented Cottage Cheese. Genes.

[42] Wang Q., Li G., Qin W., Cai J., Wang N. (2024). Evaluation of in Vitro Simulated Digestion and Fermentation Characteristics of the Crude Exopolysaccharide from *Levilactobacillus brevis* M-14. J. Food Sci..

[43] Singh N., Ghosh P.K., Chakraborty S., Majumdar S. (2021). Decoding the Pathways of Arsenic Biotransformation in Bacteria. Environ. Sustain..

[44] Montoya E.A.R., Hernández L.E.M., Escareño M.P.L. (2015). Impacto del arsénico en el ambiente y su transformación por microorganismos. Terra Latinoam..

[45] Domene A., Orozco H., Rodríguez-Viso P., Monedero V., Zúñiga M., Vélez D., Devesa V. (2024). Lactobacillus Strains Reduce the Toxic Effects of a Subchronic Exposure to Arsenite through Drinking Water. Environ. Res..

[46] De Matuoka E Chiocchetti G., Monedero V., Zúñiga M., Vélez D., Devesa V. (2020). In Vitro Evaluation of the Protective Role of Lactobacillus StrainsAgainst Inorganic Arsenic Toxicity. Probiot. Antimicrob. Proteins.

[47] Bhakta J.N., Ohnishi K., Tsunemitsu Y., Ueno D., Manna K. (2022). Assessment of Arsenic Sorption Properties of Lactic Acid Bacteria Isolated from Fecal Samples for Application as Bioremediation Tool. Appl. Water Sci..

[48] Visciano P. (2025). Arsenic in Water and Food: Toxicity and Human Exposure. Foods.

[49] Shandana, Khan A., Waqas M., Nawab J., Idress M., Kamran M., Khan S. (2024). Total Arsenic Contamination in Soil, Vegetables, and Fruits and Its Potential Health Risks in the Chitral Valley, Pakistan. Int. J. Sediment Res..

[50] Qian L., Yan B., Zhou J., Fan Y., Tao M., Zhu W., Wang C., Tu Q., Tian Y., He Q. (2024). Comprehensive Profiles of Sulfur Cycling Microbial Communities along a Mangrove Sediment Depth. Sci. Total Environ..

[51] Barton L.L., Ritz N.L., Fauque G.D., Lin H.C. (2017). Sulfur Cycling and the Intestinal Microbiome. Dig. Dis. Sci..

[52] Gänzle M.G., Follador R. (2012). Metabolism of Oligosaccharides and Starch in Lactobacilli: A Review. Front. Microbiol..

[53] Zhang Y., Li L., Pang X., Zhang S., Liu Y., Wang Y., Xie N., Li X. (2026). Lactic Acid Bacteria as the Green and Safe Food Preservatives: Their Mechanisms, Applications and Prospects. Foods.

[54] Karyani T.Z., Ghattavi S., Homaei A. (2023). Application of Enzymes for Targeted Removal of Biofilm and Fouling from Fouling-Release Surfaces in Marine Environments: A Review. Int. J. Biol. Macromol..

[55] Liang S., Wang X., Li C., Liu L. (2024). Biological Activity of Lactic Acid Bacteria Exopolysaccharides and Their Applications in the Food and Pharmaceutical Industries. Foods.

[56] Wang J., Ji H., Wang S., Liu H., Zhang W., Zhang D., Wang Y. (2018). Probiotic *Lactobacillus plantarum* Promotes Intestinal Barrier Function by Strengthening the Epithelium and Modulating Gut Microbiota. Front. Microbiol..

[57] Bengoa A.A., Dardis C., Gagliarini N., Garrote G.L., Abraham A.G. (2020). Exopolysaccharides from Lactobacillus Paracasei Isolated from Kefir as Potential Bioactive Compounds for Microbiota Modulation. Front. Microbiol..

[58] Zielińska K.J., Stecka K.M., Miecznikowski A.H., Suterska A.M. (2000). Degradation of Raw Potato Starch by an Amylolytic Strain of *Lactobacillus plantarum* C. Progress in Biotechnology.

[59] Jurášková D., Ribeiro S.C., Silva C.C.G. (2022). Exopolysaccharides Produced by Lactic Acid Bacteria: From Biosynthesis to Health-Promoting Properties. Foods.

[60] Busk P.K., Lange L. (2015). Classification of Fungal and Bacterial Lytic Polysaccharide Monooxygenases. BMC Genom..

[61] Nakamura A.M., Nascimento A.S., Polikarpov I. (2017). Structural Diversity of Carbohydrate Esterases. Biotechnol. Res. Innov..

[62] Koutsoumanis K., Allende A., Alvarez-Ordóñez A., Bolton D., Bover-Cid S., Chemaly M., Davies R., De Cesare A., Hilbert F., EFSA (2022). Update of the List of QPS-Recommended Biological Agents Intentionally Added to Food or Feed as Notified to EFSA 15: Suitability of Taxonomic Units Notified to EFSA until September 2021. EFSA J..

[63] Feichtinger M., Mayrhofer S., Kneifel W., Domig K.J. (2016). Tetracycline Resistance Patterns of Lactobacillus Buchneri Group Strains. J. Food Prot..

[64] Gueimonde M., Sánchez B., de los Reyes-Gavilán C.G., Margolles A. (2013). Antibiotic Resistance in Probiotic Bacteria. Front. Microbiol..

[65] Quaresma L.S., Santos R.C.V., Gomes G.C., Américo M.F., Campos G.M., Laguna J.G., Barroso F.A.L., Azevedo V., de Jesus L.C.L. (2024). Multidrug Resistance Profile in *Lactobacillus delbrueckii:* A Food Industry Species with Probiotic Properties. World J. Microbiol. Biotechnol..

[66] Dentice Maidana S., Aristimuño Ficoseco C., Bassi D., Cocconcelli P.S., Puglisi E., Savoy G., Vignolo G., Fontana C. (2020). Biodiversity and Technological-Functional Potential of Lactic Acid Bacteria Isolated from Spontaneously Fermented Chia Sourdough. Int. J. Food Microbiol..

[67] Reiner K. (2010). Catalase Test Protocols.

[68] Gevers D., Huys G., Swings J. (2001). Applicability of Rep-PCR Fingerprinting for Identification of Lactobacillus Species. FEMS Microbiol. Lett..

[69] Heras J., Domínguez C., Mata E., Pascual V., Lozano C., Torres C., Zarazaga M. (2015). GelJ—A Tool for Analyzing DNA Fingerprint Gel Images. BMC Bioinform..

[70] Andrews S. (2010). FastQC: A Quality Control Tool for High Throughput Sequence Data. http://www.bioinformatics.babraham.ac.uk/projects/fastqc/.

[71] Chen S., Zhou Y., Chen Y., Gu J. (2018). Fastp: An Ultra-Fast All-in-One FASTQ Preprocessor. Bioinformatics.

[72] Ewels P., Magnusson M., Lundin S., Käller M. (2016). MultiQC: Summarize Analysis Results for Multiple Tools and Samples in a Single Report. Bioinformatics.

[73] Wick R.R., Judd L.M., Gorrie C.L., Holt K.E. (2017). Unicycler: Resolving Bacterial Genome Assemblies from Short and Long Sequencing Reads. PLoS Comput. Biol..

[74] Gurevich A., Saveliev V., Vyahhi N., Tesler G. (2013). QUAST: Quality Assessment Tool for Genome Assemblies. Bioinformatics.

[75] Schwengers O., Jelonek L., Dieckmann M.A., Beyvers S., Blom J., Goesmann A. (2021). Bakta: Rapid and Standardized Annotation of Bacterial Genomes via Alignment-Free Sequence Identification. Microb. Genom..

[76] Chaumeil P.-A., Mussig A.J., Hugenholtz P., Parks D.H. (2022). GTDB-Tk v2: Memory Friendly Classification with the Genome Taxonomy Database. Bioinformatics.

[77] Meier-Kolthoff J.P., Göker M. (2019). TYGS Is an Automated High-Throughput Platform for State-of-the-Art Genome-Based Taxonomy. Nat. Commun..

[78] Zhou Z., Tran P.Q., Breister A.M., Liu Y., Kieft K., Cowley E.S., Karaoz U., Anantharaman K. (2022). METABOLIC: High-Throughput Profiling of Microbial Genomes for Functional Traits, Metabolism, Biogeochemistry, and Community-Scale Functional Networks. Microbiome.

[79] Liu Y.-Y., Hsu C.-Y., Yang Y.-C., Huang C.-H., Chen C.-C. (2023). ProbioMinServer: An Integrated Platform for Assessing the Safety and Functional Properties of Potential Probiotic Strains. Bioinform. Adv..

[80] Bai Z., Zhang N., Jin Y., Chen L., Mao Y., Sun L., Fang F., Liu Y., Han M., Li G. (2023). Comprehensive Analysis of 84 Faecalibacterium Prausnitzii Strains Uncovers Their Genetic Diversity, Functional Characteristics, and Potential Risks. Front. Cell. Infect. Microbiol..

