# Supplementary material for: Genomic Insights into the Probiotic Potential of Lactic Acid Bacteria Isolated from Tocosh: Traditional Peruvian Fermented Potatoes
Source: Int J Mol Sci. 2026 Apr 29;27(9):3981. doi: 10.3390/ijms27093981 (PMC13164450; doi:10.3390/ijms27093981)
Supplement: Supplementary file 1 [file ijms-27-03981-s001.zip › ijms-4198248-supplementary.pdf]

TABLE S1. **ASSEMBLY QUALITY REPORT**

Code	Completeness	Contamination	Coding Density	Contig N50	Average Gene Length	Genome Size	GC Content	Total Coding Sequences	Total Contigs	Max Contig Length
UNCP-C3M03	99.98	0.25	0.887	186600	294.41258	2007050	0.41	2019	40	454277
UNCP-C3M20	99.99	0.22	0.863	120201	288.637556	2444486	0.46	2439	65	360146
UNCP-C6M04	100	0.49	0.847	264923	295.934206	3357411	0.44	3207	41	556647
UNCP-C6M10	99.99	0.1	0.879	84353	286.802887	1963444	0.42	2009	77	213039
UNCP-T3M03	100	0.49	0.847	405339	296.412371	3356634	0.44	3201	39	553359
UNCP-T3M62	100	0.49	0.846	264353	296.157615	3357314	0.44	3204	40	469268
UNCP-T6M03	99.99	0.09	0.879	87917	287.358923	1964679	0.42	2006	72	213039
UNCP-T6M32	99.99	0.15	0.864	120201	290.484114	2410130	0.46	2392	61	360147
UNCP-T6M43	99.99	0.21	0.867	324637	285.774332	2770502	0.45	2805	44	398887
UNCP-H3M02	100	0.48	0.867	81738	283.142749	2576964	0.46	2634	110	382894
UNCP-H3M04	99.89	0.13	0.856	121205	290.899149	3105671	0.46	3054	108	422926
UNCP-H3M13	99.99	0.4	0.872	88566	299.661198	2509738	0.44	2438	87	284241
UNCP-H3M17	99.99	0.12	0.853	84745	296.328739	3017614	0.46	2902	97	280922
UNCP-H3M25	99.98	0.21	0.853	65427	290.850386	3042223	0.46	2981	139	311723
UNCP-H6M06	99.99	0.04	0.869	670852	294.292929	2612030	0.46	2574	13	961359
UNCP-H6M08	99.97	0.67	0.853	78047	278.62845	3223644	0.46	3297	258	291154
UNCP-H6M09	100	0.38	0.844	183662	293.192972	3375953	0.44	3244	54	707613
UNCP-H6M14	99.99	0.12	0.854	83001	296.559931	3010899	0.46	2895	94	237846
UNCP-H6M16	100	0.04	0.852	115825	291.650288	3022862	0.46	2951	88	472605
